# The Epigenetic Landscape of Vascular Calcification: An Integrative Perspective

**DOI:** 10.3390/ijms21030980

**Published:** 2020-02-01

**Authors:** Yi-Chou Hou, Chien-Lin Lu, Tzu-Hang Yuan, Min-Tser Liao, Chia-Ter Chao, Kuo-Cheng Lu

**Affiliations:** 1 Division of Nephrology, Department of Medicine, Cardinal-Tien Hospital, School of Medicine, Fu-Jen Catholic University, New Taipei City 234, Taiwan; athletics910@gmail.com; 2 Division of Nephrology, Department of Medicine, Fu-Jen Catholic University Hospital, School of Medicine, Fu-Jen Catholic University, New Taipei City 243, Taiwan; janlin0123@gmail.com (C.-L.L.); kuochenglu@gmail.com (K.-C.L.); 3 Graduate Institute of Toxicology, National Taiwan University College of Medicine, Taipei 104, Taiwan; yuan.tzu.h@gmail.com; 4 Department of Pediatrics, Taoyuan Armed Forces General Hospital, Taoyuan City 325, Taiwan; liaoped804h@yahoo.com.tw; 5 Department of Pediatrics, Tri-Service General Hospital, National Defense Medical Center, Taipei 114, Taiwan; 6 Department of Pediatrics, Fu-Jen Catholic University Hospital, School of Medicine, Fu-Jen Catholic University, New Taipei City 243, Taiwan; 7 Nephrology division, Department of Internal Medicine, National Taiwan University Hospital, Taipei 100, Taiwan; 8 Department of Internal Medicine, National Taiwan University Hospital BeiHu Branch, Taipei 108, Taiwan

**Keywords:** chronic kidney disease, diabetes mellitus, epigenetic, medial calcification, microRNA, phosphate, vascular calcification, vascular smooth muscle cells

## Abstract

Vascular calcification (VC) is an important complication among patients of advanced age, those with chronic kidney disease, and those with diabetes mellitus. The pathophysiology of VC encompasses passive occurrence of physico-chemical calcium deposition, active cellular secretion of osteoid matrix upon exposure to metabolically noxious stimuli, or a variable combination of both processes. Epigenetic alterations have been shown to participate in this complex environment, through mechanisms including DNA methylation, non-coding RNAs, histone modifications, and chromatin changes. Despite such importance, existing reviews fail to provide a comprehensive view of all relevant reports addressing epigenetic processes in VC, and cross-talk between different epigenetic machineries is rarely examined. We conducted a systematic review based on PUBMED and MEDLINE databases up to 30 September 2019, to identify clinical, translational, and experimental reports addressing epigenetic processes in VC; we retrieved 66 original studies, among which 60.6% looked into the pathogenic role of non-coding RNA, followed by DNA methylation (12.1%), histone modification (9.1%), and chromatin changes (4.5%). Nine (13.6%) reports examined the discrepancy of epigenetic signatures between subjects or tissues with and without VC, supporting their applicability as biomarkers. Assisted by bioinformatic analyses blending in each epigenetic component, we discovered prominent interactions between microRNAs, DNA methylation, and histone modification regarding potential influences on VC risk.

## 1. Introduction

Vascular calcification (VC) denotes the deposition of calcium within the vascular wall and is a major contributor to the cardiovascular risk associated with chronic kidney disease (CKD)/end-stage renal disease (ESRD), diabetes mellitus (DM), and vascular aging. The most renowned type of VC, coronary artery calcification (CAC), constitutes evidence of coronary atherosclerosis and correlates closely with the burden of vascular intimal calcification resulting from atherogenic plaques [1]. On the other hand, vascular medial calcification, or Monckeberg’s sclerosis, involves a different layer within the vascular wall and causes vessel stiffening, blood pressure elevation, cardiac hypertrophy, and adverse cardiovascular events [2]. Calcification within the vascular wall was originally perceived to result from passive physio-chemical calcium deposition with extracellular localization decades ago, but mounting evidence supports that active vascular smooth muscle cell (VSMC) trans-differentiation with osteoblast-like phenotype that carries out osteoid matrix secretion under noxious stimuli may be the major pathogenic mechanism responsible for calcification progression [3].

The coordinated interplay between various cellular components, including endothelial cells, VSMCs, fibroblasts, macrophages, and probably other immunocytes work in concert to promote calcification development within the vascular wall. Traditional wisdom regarding the VC pathophysiology centers around the up-regulation of Runt-related transcription factor 2 (RUNX2), a master upstream transcription factor of osteoblasts, and bone morphogenetic protein (BMP) in VSMCs and susceptible cells, aggravated further by persistent inflammation (cytokines), oxidative stress from advanced glycation or uremic toxin, defective osteoid resorption, and down-regulation of anti-calcific proteins or molecules [4]. However, more mechanistic research, starting since early 2010, points to the possibility that epigenetic processes serve as emerging players in the pathogenesis of VC [5]. Epigenetics describes the heritable and acquired genetic alterations that modulate the expression of one or more DNA sequences. Prior studies have already established its importance in carcinogenesis [6], metabolic dysregulation [7], and cardiovascular diseases [8]. It is then likely that epigenetic processes, including DNA methylation, histone modifications, non-coding RNAs (especially microRNAs (miRNAs)), and chromatin changes participate in the formation and perpetuation of VC. Understanding how epigenetic players shape the initiation and progress of VC can be extremely helpful for counteracting VC, for which current therapeutic regimens are frequently futile.

Existing reviews focusing on VC essentially represent the opinion pieces of field experts [9,10,11,12], but most of them fail to summarize the diverse spectrum of epigenetic processes in action for VC, are frequently process-specific, and can be outdated. To fill this knowledge gap, we conducted an updated systematic review to compile available knowledge regarding the pathogenetic and clinical implications of epigenetic processes involved in VC.

## 2. Strategy of Literature Review and Findings

We systematically searched through databases including PUBMED and MEDLINE to identify clinical, translational, and experimental reports addressing VC up to 30 September 2019, based on the following keyword combinations: “vascular calcification” or “vascular smooth muscle cells” + “calcification” AND “epigenetics”, “microRNA” or “miRNA”, “histone”, “histone deacetylase (HDAC)”, “histone methyltransferase (HMT)”, “non-coding RNA”, “DNA methylation”, “CpG”, “DNA methyltransferase (DNMT)”, “nucleosome”, or “chromatin”. Reports presenting original attempts to examine the epigenetic changes resulting from, concurrent with, and predisposing human/animal/cells to VC were retrieved, with their findings summarized. We excluded reviews, non-English reports, clinical trial protocols, articles whose focus were irrelevant to VC, and those involving non-epigenetic mechanisms. We divided the remaining reports according to their epigenetic mechanisms, including non-coding RNA, DNA methylation, histone modification, and chromatin changes; some of these reports were assigned in the category “biomarkers” if pathophysiological processes were not tested or only differences between clinical specimens were quantified.

**Figure 1:**
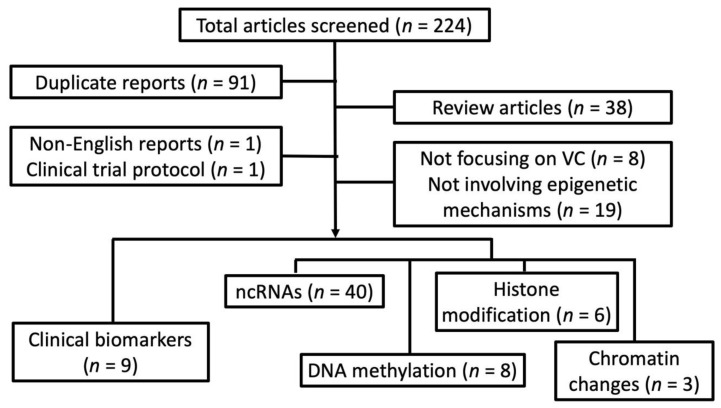
A flow chart detailing our systematic review strategy and the derivation of included reports. ncRNA, non-coding RNA; VC, vascular calcification.

After screening through 224 articles, we identified 66 original reports examining epigenetic processes in VC (Figure 1).

Interestingly, most reports (*n* = 40; 60.6%) [13,14,15,16,17,18,19,20,21,22,23,24,25,26,27,28,29,30,31,32,33,34,35,36,37,38,39,40,41,42,43,44,45,46,47,48,49,50,51,52] looked into the pathogenic role of non-coding RNA in VC, while histone modification (*n* = 6; 9.1%) [53,54,55,56,57,58], DNA methylation (*n* = 8; 12.1%) [5,59,60,61,62,63,64,65], and chromatin changes (*n* = 3; 4.5%) [66,67,68] accounted for one-fourth only. Nine (13.6%) [69,70,71,72,73,74,75,76,77] examined the discrepancy of epigenetic signatures between subjects or animals with and without VC but not their pathogenic influences. In the following sections, we summarize results from these reports and try to synthesize an inter-connected network of epigenetic regulation of VC based on the existing data assisted by bioinformatic integration.

## 3. miRNAs in VC: Positive and Negative VC Regulators

The evidence for miRNA in VC did not emerge until 2011, when Goettsch et al. first pinpointed miR-125b as a potential repressor of osteoblastic differentiation of VSMCs [13]. The number of reports addressing the influences of different miRNAs on VC rose successively over time, and the majority provide functional characterization of the index miRNA(s).

Among the miRNA studies we retrieved, three provide a global view of altered miRNA during the course of VC using the profiling approach based on VSMCs or calcified aortic explants from animals [27,43,46]. Chaturvedi and colleagues compared the miRNA expression profiles between calcified rat VSMCs and their matrix vesicles (MVs) [27]; they disclosed that MVs secreted by calcified VSMCs were enriched by 33 differentially expressed miRNAs, which were predicted to regulate VSMC contraction, differentiation, and proliferation by targeting MAP kinase, Wnt signaling, and protein phosphorylation/ubiquitination. Fakhry et al. compared the miRNA microarray results between induced calcified rat aorta and non-calcified controls and showed that 17 and 16 miRNAs were differentially expressed in calcified aortas at day 3 and 6 after calcification started, respectively [43]. These miRNAs, after being validated by individual quantitative polymerase chain reaction (qPCR), were predicted to affect inflammatory cytokine secretion, nuclear factor-κB (NF-κB) activation, apoptosis, and extracellular matrix depositions. Furthermore, miRNAs were also shown to alter significantly following successfully management of VC in experimental settings. Guo et al. tested whether miRNA expression levels change after treating calcified VSMCs with stem cell-derived exosomes using a microarray [46]; they disclosed that 63 and 1424 miRNAs significantly increased and decreased following VSMC exposed to exosomes, respectively, while pathway analyses suggested that MAP kinase, Wnt, and the mammalian target of rapamycin (mTOR) signaling were the main response elements to VC-directed treatment. However, these three studies did not look into the details of the biological action of individual miRNAs. The role of specific miRNA in regulating VC has been repeatedly illustrated. A total of 37 different miRNA species have been implicated in the pathogenesis of different types of VC, through in vitro demonstrations of their influences on VC severity when being up- or down-regulated (Table 1). 

Among them, 15 (40.5%) have been confirmed or are suspected to promote or aggravate VC in different scenarios, while 22 (59.5%) protect against VC formation or progression. Direct gene targets have been identified for 14 miRNAs with negative VC regulatory capacity, including miR-29a/29b [14], miR-29b-3p [34], miR-30b [16,47], miR-30c [16], miR-30e [25], miR-34b/34c [29,64], miR-125b [13,22] miR-133a [19], miR-135a [30], miR-182 [42], miR-204 [15,41,50], and miR-205 [21], while few direct targets have been identified for miRNAs with VC enhancement ability (miR-34a [39], miR-128-3p [48], and miR-135a-3p [17]). In the following sections, we describe the clinical features of each VC-regulating miRNA based on their propensity for positive or negative vascular influences.

### 3.1. Negative VC-Regulating miRNAs

Several miRNAs reap the widest attention with regard to their ability to repress VC, including miR-30b/30c/30e and miR-133a/133b (four reports), and miR-125b and miR-204 (three reports) (Table 1). The miR-30 family contains six members, namely miR-30a, miR-30b, miR-30c-1, miR-30c-2, miR-30d, and miR-30e, located in chromosomes 1, 6, and 8 [78]. The miR-30 family plays an integral role in regulating the development and differentiation of bone, adipose tissues, reproductive systems, blood vessels, and intestinal tissues. Specifically, pre-osteoblasts with miR-30a/30b/30c/30d overexpression were found to have lower alkaline phosphatase (ALP) activity compared to controls, hence the term “osteomiR” [79]; RUNX2 and Smad1 were identified as the direct target of miR-30a/30b/30c/30d. Through targeting DLL4, over-expression of miR-30 in endothelial cells also facilitated angiogenesis and vascular sprouting [80], supporting its role in vascular physiology. Compatible with the anti-osteoblastic differentiation action shown in other tissues, Balderman et al. identified that miR-30b/30c bound to the 3’-untranslated regions (3’-UTR) of RUNX2 and suppressed bio-mineralization in miRNA transfected human VSMCs [16]. Louvet et al. also reported that miR-30b was down-regulated during in vitro human VSMC calcification [28]. Other researchers used mouse or rat VSMC and tissues for analysis; they showed similarly that miR-30b and miR-30e over-expression alleviated VC, but the direct targets in murine VSMCs (insulin-like growth factor 2 [IGF2] and SOX9) seemed to differ from those (RUNX2 and Smad1) of human ones, and the mechanisms involved varied as well (mTOR pathway in murine cells) [25,47].

The MiR-133 family, including miR-133a and miR-133b, are predominantly expressed in muscular tissues including cardiac and skeletal muscles (“myomiRs”), participating heavily in their development and proper functions by suppressing fibrosis progression [81]. The direct targets of miR-133 reportedly include transforming growth factor (TGF)-β1 and Akt in muscle cells [82], both of which are also involved in the pathogenesis of VSMC calcification. Indeed, Liao et al. first showed that miR-133a overexpression in mouse VSMCs ameliorated calcification severity by directly down-regulating RUNX2 levels [19], while Panizo and colleagues derived the same findings involving miR-133b in rat cells and uremic calcified aortas [32]. This is also true in human VSMCs, as shown by the decreased expression of miR-133a in calcified cells compared to non-calcified ones [28]. However, Wang et al. observed increased miR-133b expression in calcified rat aortas relative to the control [68], which was contradictory to findings of miR-133 in the non-VSMC and the VSMC experiments. This may be related to the differences in normalization controls, the status of adaptive responses, and the timing of RNA sampling during the VC process.

MiR-125b has long been deemed an onco-miR and tumor suppressive miR through targeting cell survival and apoptosis-related molecules, including p53, Bcl/Bak, and PI_3_K/Akt pathways [83]; the increased expression of miR-125b was reported in multiple types of human malignancy. In vascular tissues, miR-125b over-expression leads to a lower degree of endothelial and VSMC proliferation, more vaso-relaxation, but a greater severity of inflammation, as summarized in a prior systematic review [83]. In terms of its effect on VC, the up-regulation of miR-125b is associated with less calcium deposition by murine and human VSMCs by directly repressing Ets-1 and osterix, an important osteoblastic marker [13,22]; furthermore, our previous work demonstrated that higher VC severity among uremic patients correlates with lower circulating miR-125b levels, supporting its anti-VC effect and its clinical applicability [33].

MiR-204 was initially known for its regulatory activity toward retinal pigmented epithelia development, adipogenesis from mesenchymal stem cells (MSCs), and its anti-proliferative effect among pulmonary artery smooth muscle cells in pulmonary artery hypertension [84]. It has been shown that miR-204 promotes adipogenesis while it suppresses osteogenesis through direct inhibition of RUNX2 translation in MSCs and bone marrow stem cells [85]. A similar scenario was observed in VSMCs; Cui et al. showed that down-regulating miR-204 contributed to the development of VC in murine cells and calcified aortas through RUNX2 suppression [15], while Wang et al. further validated this relationship in human samples [50]. Lin and colleagues additionally showed that miR-204 could also oppose the progression of VC by targeting DNA methyltransferase 3a (DNMT3a) and altering DNA methylation status [41]. These findings strongly support that miR-204 serves as a potentially negative mechanism for counteracting VC.

Apart from those with multiple reports of their VC-attenuating ability, in a single report, several miRNAs exhibited a similar influence (Table 1). MiR-21, for example, a widely known onco-miR that increases in nearly most cancer types, also serves as a “mechano-miR” up-regulated in endothelial cells subjected to shear stress with anti-apoptosis ability [86]. Han et al. found that VSMC with teniposide-induced up-regulation of miR-21 had a less proliferative phenotype compared to controls, tempering the VSMC propensity to calcify [40]. MiR-25, also an onco-miR, was reported to interfere with calcium handling, attenuate inflammation, and decreased apoptosis in myocardial and renal tissues [87]. Zhang and colleagues showed that in mouse VSMCs, over-expression of miR-25 could ameliorate exogenous stimuli-triggered apoptosis and potentially subsequent calcification [51]. MiR-26a plays a pivotal role in different types of cancers by regulating cellular proliferation and participates in cardiovascular pathophysiology by targeting glycogen synthase kinase (GSK)-3β, a Wnt/β-catenin pathway component [88]. It is likely that a similar event occurs in VSMC, leading to calcification reduction, as was shown by Wu et al. in their in vitro experiments [35]. MiR-135a affect cancer progression and non-cancer cell physiology by regulating inflammatory signaling by negatively affecting the NF-κB pathway and PI_3_K-R2 expression [89]. Lin et al., extrapolating from results in other tissues, found that VSMCs with miR-135a inhibition had more severe calcification upon high phosphate (HP) exposure, through Kruppel-like factor 4 (KLF4) and STAT3 up-regulation [30]. MiR-142-3p was previously known to maintain stem cell pluripotency, suppress cardiac hypertrophy, and assist in lung development [90]. Ketszeri and colleagues showed that uremic patients had lower circulating miR-142-3p levels and poor vasorelaxation capacity, with similar results shown in uremic rat aortas [45]. Different from the above miRNAs, miR-143-5p/3p are well characterized miRNAs in vascular biology and regulate VSMC contractility, differentiation, and phenotypic switching [91]. Louvet et al. disclosed that miR-143-3p was down-regulated during VSMC calcification but reversed by VC-counteracting treatments [28]. MiR-182, a member of the miR-183 family, has been shown to suppress FoxO1 expression, causing impaired osteoblast differentiation and retarded osteogenesis in MSCs [92]. Zhang et al. showed that a similar phenomenon occurred in VSMCs, in which miR-182 over-expression led to reduced calcium deposition [42]. MiR-205 exhibits diverse influences in cancer and non-cancerous tissues by targeting multiple oncogenes and growth factors. Qiao and colleagues further demonstrated that miR-205 attenuated human VSMC calcification by inhibiting RUNX2 expression [21]. MiR-211 reportedly plays a role in chondrocyte differentiation by decreasing inflammatory cytokine secretion and matrix degradation [93], and Panizo et al. showed that miR-211 regulated VC in vitro and in vivo, although the mechanism involved was not shown clearly [32]. MiR-297a, previously not reported to be expressed in VSMCs, was also shown to suppress VC in a nicotine and vitamin D-induced VC rat model, although more evidence is still needed [31]. MiR-302b, a well-known oncogene in multiple tumors, ameliorated VC through direct down-regulation of RUNX2 [38]. The above findings suggest that multiple miRNAs may exert anti-VC actions in VSMCs; however, evidence for other potential pathways involved is still unclear and remains an active area of investigation.

### 3.2. Positive VC-Regulating miRNAs

Anecdotal reports suggested that certain miRNAs could promote the progression of VC as well, although the number of such reports is significantly fewer than that of negative VC regulating miRNAs. Through miRNA microarray analysis, Liu et al. discovered that miR-32 increased RUNX2 expression and promoted VC in VSMCs by directly targeting phosphatase and tensin homolog (PTEN) [37], which was also found in cancer cells including colorectal cancer and MSCs [94]. MiR-128-3p has been shown to suppress inhibitors of Wnt/β-catenin and TGF-β signaling in cancer cells [95], leading to canonical Wnt activation and tumor resistance; Wang et al. demonstrated that pancreatic tissue with miR-128-3p up-regulation also accelerated VC by intensifying Wnt/β-catenin signaling in diabetic animals, not VSMCs [48]. In endothelial cells, miR-135a-3p potently antagonizes angiogenesis and potentially serves as a tumor suppressor [96], while Gui et al. additionally showed that VSMCs with miR-128-3p overexpression reduced Ca^2+^ efflux and became calcified [17]. MiR-155 is extensively involved in diseases including malignancy, inflammatory, and cardiovascular disorders due to its wide signaling network connection [97]. Wang and colleagues similarly disclosed that calcified VSMCs exhibited increased expression of miR-155 compared to control cells, although the direct target was unclear [68]. MiR-221/222 are homologous miRNAs that modulate cell growth, inflammation, and oxidative stress, and over-expression of both miRNAs in VSMCs assists in calcification progression [20]. Finally, several miRNAs, including miR-712-3p, miR-714, miR-762, miR-2861, and miR-3960 have been found to modulate aortic calcification severity by reducing Ca^2+^ efflux and altering histone acetylation status [17,24]. It is interesting to observe that very few of these positive VC regulating miRNAs have their direct targets identified (Table 1), and this phenomenon may hint at the possibility that positive VC regulating miRNAs are potential therapeutic targets to be considered in future studies.

### 3.3. MiRNAs with Controversial Influences on VC

Three groups of miRNAs, namely miR-29, miR-34 family, and miR-223, exhibited pro- and anti-calcification properties in different reports (Table 1). The MiR-29 family contain three members, namely miR-29a, 29b, and 29c; these members exhibit immunoregulatory ability, affect muscle cell physiology, and modulate cell survival [98]. Experiments based on rat, human VSMCs, and calcified arteries from rat showed that miR-29a/29b/29c could ameliorate [14,34,44] or aggravate [26,32] calcification progression (Table 1). We believe that this controversial finding can be interpreted from the following perspective; first, those with the direct targets of miR-29 family members uncovered [14,34] all concluded that these miRNAs were negative VC regulators. Among reports concluding miR-29 family members are pro-calcific, Sudo et al. showed that VSMCs with miR-29b over-expression and silencing had nearly 30% and 35% higher and lower calcium deposition than control, respectively, while the miR-29a function was unclear [26]. Panizo et al. showed that silencing miR-29b could lead to less severe calcification but over-expressing miR-29b did not exhibit a corresponding pro-calcific effect [32]. On the other hand, reports that support an anti-calcific role of miR-29 family members uniformly identify a compatible trend of calcification changes upon up- and down-regulation of miR-29a/29b [14] and miR-29b-3p [34]. Consequently, we believe that miR-29 family members more likely ameliorate calcification severity than aggravate calcification in light of current evidence.

The MiR-34 family, including miR-34a/34b/34c, is a prototypic tumor suppressor that represses cancer cell viability and metastasis and is regulated by p53 [99]. Over-expression of miR-34 family members in other cell types may modulate their differentiation, apoptosis, and self-renewal properties. In VSMCs, miR-34b/34c has been shown to attenuate VC severity through down-regulating special AT-rich sequence-binding protein 2 (SATB2) and subsequently RUNX2 expression levels [29], while down-regulating miR-34b in VSMCs also leads to enhanced calcification [64]. On the contrary, Badi et al. focused on the role of miR-34a in the pathogenesis of VC using miR-34 knockout mice [39]. They found that such genetically manipulated mice receiving vitamin D had less soft tissue and aortic calcification compared to wildtype littermates, and the effect was mediated through miR-34-dependent sirtuin 1 (SIRT1) suppression. Such discrepancy regarding the biologic effect within the miR-34 family may be related to differential targeting of each member. For example, miR-34a but not miR-34b/34c has been reported to inhibit SIRT6 expression in several cell lines, and SIRT6 is a renowned inhibitor of vascular aging and atherosclerosis [100]. Despite all members of miR-34 family being shown to inhibit canonical Wnt signaling, differences regarding their efficacy to suppress Wnt pathway members still vary [101]. Along this line, different miR-34 members may have their own balance between pro-calcific and anti-calcific signaling, leading to the observed differences in their influences on VC.

MiR-223 was first characterized by its abundant expression in the hematopoietic system and its ability to induce granulopoiesis, but its role in systemic and local immunoregulation and pro-inflammation has gradually been recognized [102]. By virtue of its suppressive effect toward IκB kinase α (IKKα), STAT3, and NOD-, LRR- and pyrin domain-containing protein 3 (NLRP3), aberrant miR-223 levels are associated with various inflammatory disorders, diabetes mellitus with insulin resistance, and atherosclerosis [102,103]. However, a cell specific effect is evident regarding miR-223 in the pathogenesis of VC. M’Baya-Moutoula et al. revealed that in monocyte/macrophage cell lines, miR-223 over-expression facilitated their differentiation toward osteoclasts and potentially ameliorated the degree of calcification within the vessel wall [23]. Similar findings were obtained by Li et al., although the target gene differed [36]. Alternatively, VSMCs with miR-223 over-expression had increased proliferation and migratory ability, while calcified VSMCs had significantly up-regulated miR-223 levels [18]. Cavallari et al. also showed that uremic patients with VC had nearly 2-fold higher levels of plasma exosomal miR-223 compared to control groups [49], lending support to the potential agonistic effect for VC for miR-223. This difference in miR-223 effect on VC seems to depend on cell type and the clinical context being evaluated.

### 3.4. Non-Coding RNA and VC

We only found one report addressing the influence of long non-coding RNA (lncRNA) on VSMC calcification. Song et al. discovered that a lncRNA, H19, was down-regulated in an in vitro model of VC, which was reversible upon VC counteracting treatments [52]. However, how this lncRNA regulates the VC process has not been shown.

### 3.5. An Integrative Perspective: Other Targets of Literature-Identified miRNAs in VC

**Figure 2:**
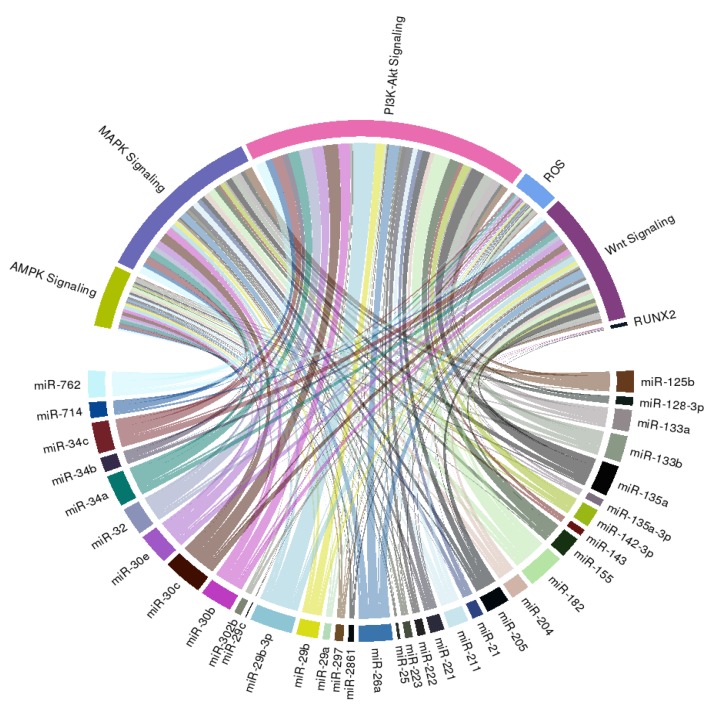
A circular ribbon chart depicting interactions between literature-identified miRNAs in Table 1 and known molecular mediators of vascular calcification. The thickness of the ribbon is reflective of the scores assigned by the literature addressing the relationship based on miRNAtap (R package version 1.16.0; R foundation for Statistical Computing, Vienna, Austria) and topGO (R package version 2.34.0; R foundation for Statistical Computing, Vienna, Austria).

In order to extend the spectrum of potential downstream effectors of miRNAs we summarized above, we further construct a circular ribbon plot, examining the complex inter-relationship between candidate miRNAs (Table 1) and pathways/signaling involved in VC as reported by others (Figure 2).

We discovered that an intimate relationship exists between these miRNAs and PI_3_K-Akt, MAPK, AMPK, and Wnt signaling, lending support to the possibility that multiple pathways are involved in the miRNAs regulation of VC probability, besides those reported by studies included in this systematic review. We additionally found that many candidate miRNAs influence reactive oxygen species generation or anti-oxidant signaling, both of which are also critical mediators and propagators of VC (Figure 2). Few of these miRNAs directly target RUNX2, the master osteoblastic differentiation gene, which is compatible with our summary of the existing literature (Table 1).

## 4. DNA Methylation in VC

A total of eight articles evaluate the influence of DNA methylation in the process of VC, ranging from in vitro to in vivo settings [5,59,60,61,62,63,64,65]. Common precipitators of VC, including HP environment, uremic toxin (indoxyl sulfate (IS)), and extracellular matrix constituents, have all been found to intensify the severity of VC through changes in methylation status of specific genes involved in osteoblastic differentiation. For example, HP exposure in vitro is a powerful inducer of calcification for treated VSMCs, by inducing DNMT activity and SM22α promoter methylation [5]. It is interesting to observe that DNMT inhibition using 5-aza-2’-deoxycytidine among HP-treated VSMCs similarly promotes calcification progression by demethylating the promoter region of alkaline phosphatase [59]. HP further causes the up-regulation of methyl-CpG binding protein 2 (Mecp2) and suppresses the expression of peroxisome proliferator-activated receptor-γ (PPAR-γ) and Klotho, a calcification antagonizer, among VSMCs [63]. Apart from the methylation of calcification inhibitors, HP also contributes to VSMC calcification via DNMT3a up-regulation and hyper-methylation of the upstream promoter of miR-34b, and such methylation change is reversible upon 5-aza-2’-deoxycytidine use [64].

Other calcification stimuli also participate in the pathogenesis of VC through methylation changes. Chen et al. reported that IS treatment enhanced the upstream CpG methylation status of Klotho in treated VSMCs, thereby lowering Klotho expression and precipitating VSMC calcification [60]. Such tendency for hypermethylation might be related to VSMC DNMT1 and DNMT3a up-regulation and could be improved by 5-aza-2’-deoxycytidine. In addition, Xie et al. showed that stiffening of VSMC extracellular matrices using polyacrylamide gel caused intracellular DNMT1 down-regulation, facilitating the osteogenic trans-differentiation of VSMC by altering the methylation status of SM22α and α-smooth muscle actin (SMA) [61].

In addition to DNMT-mediated gene expression changes, protein arginine N-methyltransferase (PRMT)-mediated changes may also play a role in the pathogenesis of VC, mostly through modulating non-canonical Wnt signaling. Cheng et al. showed that in low density lipoprotein receptor (LDLR) knockout mice, conditional LRP6 deletion in VSMCs activated the non-canonical Wnt pathway by altering PRMT-1 activity and ALP promoter activities [65]. Ramachandran and colleagues similarly disclosed that in LRP6 knockout mice, monomethylation of 21 genes increased in VSMCs, accompanied by reductions in demethylation level compared to wild type littermate [62]. Specifically, the enhanced monomethylation of G3BP1 in LRP6 deficient VSMCs is linked to nuclear factor of activated T cells-3c (NFAT3c) up-regulation and elevating alkaline phosphatase transcription, predisposing it to the formation of VC.

## 5. Histone Modification in VC

Another important mechanism for epigenetic regulation is histone modification, consisting of the methylation, acetylation, phosphorylation, ubiquitination, and sometimes sumoylation of histone, causing chromatin conformational and localized gene expression changes. Histone deacetylation and methylation related to HDAC and HMT activities are two of the most common processes involved. Based on results from the six reports we identified (Figure 1), we try to describe the biologic importance of histone modification in VC.

The role of histone modification in the pathogenesis of VC was first shown by Azechi et al., who found that the inhibition of HDAC using trichostatin A increased calcium deposition of HP-treated VSMCs by up-regulating ALP expression [53]. Another group subsequently showed that HDAC1 inhibition due to MDM2 action or chemicals such as apicidin also contributed to VC aggravation in vitro and in vivo, and proteasomal degradation of HDAC appeared to be the main mechanism [54]. On the contrary, Abend and colleagues reported that HDAC4 was up-regulated early during the course of in vitro calcification, and the pharmacological inhibition of the upstream regulators of HDAC4 could reduce VC severity [56]. Histone methylation changes also occur in calcified VSMCs. Kurozumi et al. examined an in vitro model of interleukin-6 (IL-6)-induced VSMC calcification, and discovered that JMJD 2B, a histone lysine demethylase, modulated the expression of RUNX2 through a transcriptional repressor, H3K9me3, and a transcriptional enhancer, H3K4me3, located in the RUNX2 promoter region [57]. Chen et al., alternatively, disclosed that down-regulation of lysine methyltransferase 7/9 (SET7/9) played a role in another model of IS-induced VSMC calcification [58]. However, the exact methylation signature in the IS model was not described clearly in their findings.

Histone modifications also occur in other cellular contributors in VC; Chinetti-Gbaguidi et al. revealed that macrophages near the calcification centers within the vascular wall had a signature of an increased H3K27me3, indicating the transcriptional repression of genes involved in osteoclast activity and bone resorption [55]. This phenotype of defective calcium recycling likely contributes to the propagation of calcification lesions within the vascular intima and media, although more evidence is needed to gauge the extent to which macrophage with osteoclastic differentiation affects the calcification process.

## 6. Chromatin Changes in VC

Relatively few reports address the chromatin changes in the process of VC, and the contribution of chromatin conformational changes to VC pathogenesis remains largely an uncharted sea. We found three reports mentioning changes in chromatin structure pertaining to VC (Figure 1): in MSCs exposed to HP, Fujita et al. observed that viable VSMCs that calcified exhibited chromatin condensation and/or fragmentation while non-calcified cells did not [66]. SMARCA, a chromatin remodeler influencing chromatin structure and gene expression, has been shown to increase significantly in calcified rat aortas compared to non-calcified ones, and SMARCA upregulation might contribute to aberrant levels of VC-regulating miRNAs [68]. Gilham and colleagues demonstrated that molecules inhibiting chromatin modification site readers such as BET protein might help improve VC, but the findings were limited to in vitro only [67]. Although results from these three studies lend support to the notion that chromatin changes can be a unique signature of VC and even be pathogenic and druggable, more mechanistic research is expected to shed light on the specificity and the efficacy of such chromatin-oriented interventions for VC.

## 7. The Cross-Talk between Epigenetic Mechanisms in VC

Among the 67 reports we included in this review, four have gone further to address the interactions between different epigenetic mechanisms involved in VC pathology [24,41,64,68]. A cross-talk between miRNAs and DNA methylation machinery has been examined by two reports [41,64], while that between miRNAs and histone modification [24] or chromatin changes [68] is also noted in one report for each. For example, a reciprocal relationship between miR-204, a negative VC regulator, and DNMT3a, a putative promoter of VC, was described in a HP-induced VSMC calcification model [41]. Expression levels of miR-34b, another negative VC regulator, are reportedly under the control of DNMT3a as well, and the inhibitory effect of DNMT3a can be salvageable through demethylating agents [64]. MiRNAs can be potential upstream regulators of HDAC, leading to calcification severity modulation, as exemplified by Xia et al. [24].

**Figure 3:**
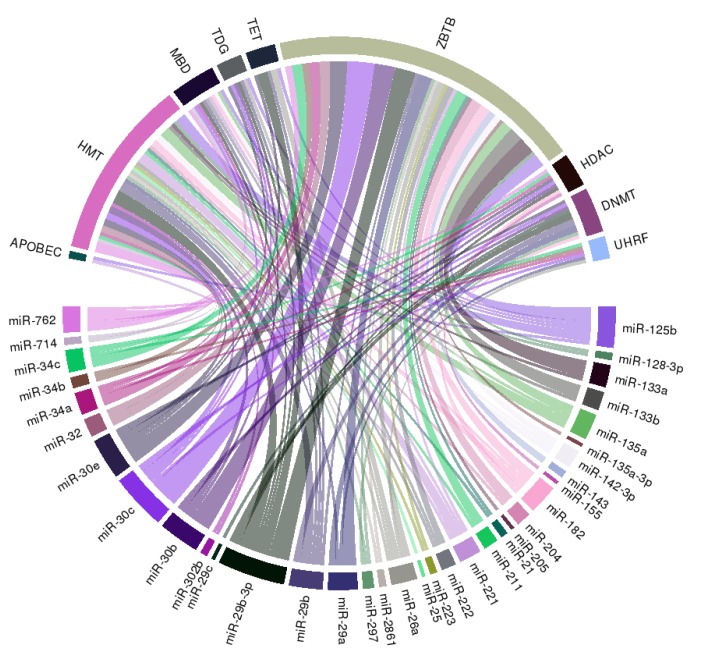
A circular ribbon chart depicting interactions between literature-identified miRNAs in Table 1, and key molecules involved in DNA methylation and histone modification. The thickness of the ribbon is reflective of the scores assigned by the literature addressing the relationship based on miRNAtap (R package version 1.16.0; R foundation for Statistical Computing, Vienna, Austria) and topGO (R package version 2.34.0; R foundation for Statistical Computing, Vienna, Austria).

We further constructed a circular ribbon plot to examine any potential regulatory relationship between miRNAs, histone modification, and DNA methylation regarding the pathogenesis of VC to better delineate the spectrum of interactions (Figure 3).

The literature-identified miRNAs (Table 1) exhibit wide connections with multiple integral components of DNA methylation, including DNMT, UHRF family, which adapt DNMT to methylated regions, TET family demethylases, TDG family, and the methyl CpG binding domain (MBD) protein family (Figure 3). Besides, we also observe wide-spread links between selected miRNAs and histone or chromatin modification related genes including HMT and HDAC families, and ZBTB family, which recruit other histone modification enzymes to chromatin, suggesting that miRNAs may serve as pivotal anchors coordinating between different epigenetic machineries in the pathogenesis of VC.

## 8. Discrepancies between Calcified and Non-Calcified Tissues or Cells: Potential for Uncovering Clinical Biomarkers

There are nine studies examining solely the differences regarding individual epigenetic machinery between calcified and non-calcified specimens [69,70,71,72,73,74,75,76,77]. Differences in circulating miRNAs between plaques with and without calcification, and between patients with and without VC are the most frequently examined topics (seven reports), followed by differences in DNA methylation status of peripheral leukocytes (two reports). These studies identified that individuals with ESRD or at risk of developing VC had significantly lower circulatory miR-15b [69], miR-223-3p and miR-93 [71], miR-125b [75], lower intra-plaque miR-1-3p, miR-133b, and miR-4530 [73], and higher circulatory miR-29b [74] compared to nonuremic or non-calcified ones. Other plausible VC-regulating miRNAs listed in Table 1 have not been examined in the existing literature. Specifically, only circulatory miR-125b and intra-plaque miR-133b expression levels are consistent with regard to their effect on VC across multiple different studies, as shown in Table 1, while the other miRNAs (miR-15b, miR-223-3p, miR-93, miR-1-3p, and miR-4530) have not been tested in experimental settings about their influences on VC. The utility of these putative biomarker miRNAs in clinical settings thus remains controversial.

On the other hand, Pickering et al. reported that serum miR-26a, miR-34a, and miR-223 did not differ between those with and without VC progression [77], findings that are in contrast to those by Cavallari et al., who described that plasma miR-223 increased in those with ESRD under chronic dialysis [49]. It is likely that the numbers of subjects being tested, the timing of the assay for circulatory miRNAs, the controls used to derive miRNA data, and the biologic fluid assayed may account for the discrepancy we observed. Optimal standardization of circulatory miRNA measurement and test setting selection is needed for a better comparison of results between reports. Finally, gender-specific differences in circulatory miRNA profiles among individuals with different VC severity may exist; Dudink and colleagues showed that male individuals with CAC had higher levels of plasma miR-103, miR-125a, miR-221, and miR-223 than those without, while female individuals with CAC and descending aorta calcification had higher levels of plasma miR-221 and miR-212, respectively [76]. Nonetheless, more data are still needed to ascertain whether gender-specific differences in epigenetic biomarkers of VC exist clinically.

## 9. Conclusion and Future Perspectives

Throughout the development, consolidation, and progression of VC, epigenetic machineries play an important regulatory role through miRNAs, DNA promoter methylation, and chromatin and histone modification, individually or in combination. MiRNAs are the most widely reported epigenetic mechanism through which VSMCs acquire osteoblast-like phenotypes when they are exposed to noxious and pro-calcific stimuli. Assisted by the up- or down-regulation of DNMT and the alteration of PRMT, calcification inhibiting genes such as negative VC-regulating miRNA may be hypermethylated, accompanied by the activation of vital osteoblast-differentiation genes in VSMCs, culminating in the occurrence of VC. Specific histone tail methylation or the deregulation of different HDAC members similarly predispose VSMCs to calcification, and histone modification further influences other cellular players in the VC foci such as macrophages. Changes in chromatin conformation and structures and their precipitators are promising contributors to VC, although the supporting evidence is still scant. The cross-talk between different epigenetic machineries regarding VC, after all, may be the ultimate realm that necessitates our attention, and there are already pilot studies addressing the biologic plausibility and pathogenic importance of these relationships. We believe that incoming studies in the near future will sharpen or even revolutionize our view on the pathogenesis of VC through an eye-opening window.

## Figures and Tables

**Table 1:** MicroRNAs involved in VC.

miRNA Species	VC Models	VC Agonistic or Antagonistic	Molecular Influences	Reference
miR-21	Human ASMC	Antagonistic	Down-regulate OPN	[40]
miR-25	Primary mouse ASMC	Antagonistic (potentially)	Down-regulate MOAP1	[51]
miR-26a	Human VSMC	Antagonistic	Down-regulate CTGF and RANKL	[35]
miR-29a/29b/29c, miR-29b-3p	Rat VSMC, uremic rat arteries, uremic patient arteries	Antagonistic	Down-regulate ADAMTS-7 (direct target, miR-29a/b)	[14]
	Human VSMC	Agonistic	Down-regulate elastin	[26]
	Primary rat ASMC, Nephrectomized rat with VC	Agonistic	Down-regulate HDAC4, CTNNBIP1, and ACVR2A	[32]
	Rat VSMC, rat calcified arteries	Antagonistic	Down-regulate MMP2 (direct target, 29b-3p)	[34]
	Human ASMC	Antagonistic	Down-regulate Wnt7b/ß-catenin	[44]
miR-30b	Human coronary SMC, human calcified coronary arteries	Antagonistic	Down-regulate RUNX2 (direct target)	[16]
	Human ASMC	Antagonistic (potentially)		[28]
	Rat VSMC, nephrectomized rat with VC	Antagonistic	Down-regulate SOX9 (direct target), up-regulate MMP, autophagy and mTOR	[47]
miR-30c/30e	Human coronary SMC	Antagonistic	Down-regulate RUNX2 (direct target, 30c)	[16]
	Mouse ASMC, ApoE KO mouse aorta	Antagonistic	Down-regulate IGF-2 (direct target, 30e)	[25]
miR-32	Mouse ASMC, OPG KO mouse, plasma from human with CAC	Agonistic	Up-regulate RUNX2, BMP2, OPN, and ALP	[37]
miR-34a/34b/34c	Aldosterone-treated rat VSMC	Antagonistic	Down-regulate SATB2 (direct target, 34b/34c)	[29]
	miR-34a KO mice	Agonistic	Down-regulate SIRT1 and Axl (direct target, 34a)	[39]
	Rat VSMC, Nephrectomized rat with VC, uremic calcified renal arteries	Antagonistic	Down-regulate Notch1 (direct target, 34b)	[64]
miR-125b	Human coronary SMC, ApoE KO mouse calcified aorta	Antagonistic	Down-regulate osterix (direct target)	[13]
	Primary rat ASMC	Antagonistic	Down-regulate Ets1 (direct target)	[22]
	Rat ASMC, adenine-feeding CKD rat with VC, sera from uremic patients	Antagonistic	Down-regulate RUNX2 and osteocalcin	[33]
miR-128-3p	Type 2 diabetic rats	Agonistic	Down-regulate ISL1 (direct target), up-regulate Wnt-1/ß-catenin and GSK-3ß	[48]
miR-133a/133b	Primary mouse VSMC	Antagonistic	Down-regulate RUNX2 (direct target, 133a), osteocalcin, and ALP	[19]
	Human ASMC	Antagonistic (potentially)		[28]
	Primary rat ASMC, Nephrectomized rat with VC	Antagonistic	Down-regulate RUNX2	[32]
	Rat ASMC, adenine-feeding rat with VC	Agonistic (potentially)		[68]
miR-135a, miR-135a-3p	Mouse ASMC, Klotho KO mouse aorta	Agonistic	Down-regulate NCX1 (135a-3p)	[17]
	Primary rat ASMC	Antagonistic	Down-regulate KLF4 (direct target) (135a)	[30]
miR-142-3p	DAB/2 mouse aorta, sera from uremic patients	Antagonistic (potentially)		[45]
miR-143	Human ASMC	Antagonistic (potentially)		[28]
miR-155	Rat ASMC, adenine-feeding rat with VC	Agonistic (potentially)		[68]
miR-182	Rat ASMC, Calcified arteries from VitD-treated rat	Antagonistic	Down-regulate SORT1 (direct target)	[42]
miR-204	Mouse ASMC, VitD-treated mouse aorta	Antagonistic	Down-regulating RUNX2 (direct target)	[15]
	Mouse VSMC, Nephrectomized mouse with VC, calcified renal arteries from uremic patients	Antagonistic	Down-regulating DNMT3a (direct target)	[41]
	Rat VSMC, adenine-feeding rat with VC, renal arteries from uremic patients	Antagonistic	Down-regulating RUNX2 (direct target)	[50]
miR-205	Human ASMC	Antagonistic	Down-regulating RUNX2 and Smad1 (direct target)	[21]
miR-211	Primary rat ASMC, Nephrectomized rat with VC	Antagonistic	Down-regulate RUNX2	[32]
miR-221/222	Primary mouse VSMC	Agonistic	Up-regulate ENPP1, PiT-1	[66]
miR-223	Human primary VSMC, ApoE KO mouse aorta	Agonistic (potentially)	Down-regulate Mef2c, RhoB	[18]
	RAW264.7 cells	Antagonistic	Up-regulate osteoclastogenesis-related genes	[23]
	RAW264.7 cells	Antagonistic	Down-regulate NF1A, RhoB	[36]
	Human VSMC, plasma from uremic patients	Agonistic	-	[49]
miR-297a	VitD-treated rat with VC	Antagonistic	Down-regulate FGF-23	[60]
miR-302b	Nephrectomized rat with VC	Antagonistic	Down-regulate BMP-2, RUNX2, Osterix	[38]
miR-712-3p	Mouse ASMC, Klotho KO mouse aorta	Agonistic	Down-regulate NCKX4	[17]
miR-714	Mouse ASMC, Klotho KO mouse aorta	Agonistic	Down-regulate PMCA1	[17]
miR-762	Mouse ASMC, Klotho KO mouse aorta	Agonistic	Down-regulate NCX1	[17]
miR-2861	Mouse primary ASMC	Agonistic	Down-regulate HDAC5	[24]
miR-3960	Mouse primary ASMC	Agonistic	Down-regulate HOXA2	[24]

ADAMTS, a disintegrin and metalloproteinase with thrombospondin motifs; ALP, alkaline phosphatase; ASMC, aortic smooth muscle cell; BMP, bone morphogenetic protein; CTGF, connective tissue growth factor; CTNNBIP, catenin beta interacting protein; DNMT, DNA methyltransferase; ENPP, ectophosphodiesterase/nucleotide phosphohydrolase; FGF-23, fibroblast growth factor-23; GSK, glycogen synthase kinase; HDAC, histone deacetylase; IGF, insulin-like growth factor; KO, knockout; MMP, metalloproteinase; MOAP, modulator of apoptosis; mTOR, mammalian target of rapamycin; NCKX, Na^+^/Ca^2+^–K^+^ exchanger; NCX, Na^+^–Ca^2+^ exchanger; OPN, osteopontin; PMCA, plasma membrane Ca^2+^ ATPase; RANKL, receptor activator of nuclear factor kappa-B ligand; VC, vascular calcification; VSMC, vascular smooth muscle cell.

## Abbreviations

3’-UTR	3’-untranslated region
ALP	alkaline phosphatase
BMP	bone morphogenetic protein
CAC	coronary artery calcification
CKD	chronic kidney disease
DM	diabetes mellitus
DNMT	DNA methyltransferase
ESRD	end-stage renal disease
GSK	glycogen synthase kinase
HDAC	histone deacetylase
HMT	histone methyltransferase
HP	high phosphate
IGF	insulin-like growth factor
IS	indoxyl sulfate
KLF	Kruppel-like factor
LDLR	low density lipoprotein receptor
lncRNA	long non-coding RNA
MBD	methyl-CpG binding domain
Mecp	methyl-CpG binding protein
miRNA	microRNA
MSC	mesenchymal stem cell
mTOR	mammalian target of rapamycin
MV	matrix vesicle
NFAT	nuclear factor of activated T cells
NF-κB	nuclear factor-κB
PPAR-γ	peroxisome proliferator-activated receptor-γ
PRMT	protein arginine N-methyltransferase
PTEN	phosphatase and tensin homolog
qPCR	quantitative polymerase chain reaction
RUNX2	Runt-related transcription factor 2
SATB	special AT-rich sequence-binding protein
SIRT	sirtuin
SMA	smooth muscle actin
TGF	transforming growth factor
VC	vascular calcification
VSMC	vascular smooth muscle cell
